# Complete Theoretical and Practical Treatise on Diseases of the Liver

**Published:** 1841-07

**Authors:** 


Art. V.

1. *Traité Complet, Théorique et Pratique, des Maladies du Foie.* Par Aug. Bonnet, Docteur en Médecine de la Faculté de Paris, &c. &c. New Edition, corrected and augmented.—*Paris*, 1841. 8vo, pp. 396.
*Complete Theoretical and Practical Treatise on Diseases of the Liver.* By Aug. Bonnet, m.d., of the faculty of Paris, &c. &c.—*Paris*, 1841.
2. *A Practical Treatise on the Diseases of the Liver and Biliary Passages.* By William Thomson, m.d., Fellow of the Royal Colleges of Physicians and Surgeons, and one of the Physicians to the Royal Infirmary of Edinburgh.—*Edinburgh*, 1841. 8vo, pp. 306.
3. *The Madras Quarterly Medical Journal.* Edited by Samuel Rogers, Assistant Surgeon. (Vol. II, for Jan., April, July, Oct. 1840.) —*Madras*, 1840. 8vo, pp. 462.

It is not unusual to find an author manifesting a disproportionate anxiety to establish a claim to originality in some theoretical view involving no practical consequence of any moment, while, in the opinion of the reader, he might, if disposed to congratulate himself, have selected other and better grounds. We think the foregoing remark applicable to M. Bonnet, who challenges consideration to himself for certain views which he alleges to be new, but which we think are not materially so, and which, granting them to be new, are certainly less important than M. Bonnet seems to suppose them. Fortunately his work possesses other and more solid grounds of reputation.

After some "Preliminary Observations," in which are given, with brevity and perspicuity, the Anatomy and Physiology of the Liver, the proper subject of the work commences, and the first chapter is devoted to the "Semeiology of Irritation of the Liver."

M. Bonnet sets out with stating that there are cases in which irritation of the liver is so slight as not to amount to a phlegmasia. He thinks that this stage or phase of hepatic derangement has been hitherto overlooked by authors, who, as he supposes, never recognize hepatic derangement unless as it presents itself *after* having merged, in a greater or less degree, into Hepatitis. He claims to be the first who has detected and drawn the characters of hepatic irritation in this its incipient form, while uncomplicated with inflammation.

We shall be better able to judge whether M. Bonnet's claim to originality on this point be well founded after having seen what, according to him, constitutes "the first degree of hepatic irritation." He owns the diagnosis to be difficult, but states it to be as follows: It being granted, he says, that an increase in the functions of an organ proves the amount of vitality in that organ to be beyond the normal degree (an inference which we for our parts are far from assenting to), one cannot but admit that a more than usually abundant secretion of bile denotes that the liver is the subject of irritation. Whenever, therefore, we find an individual with a bitter taste in the mouth, yellow and furred tongue, vomiting of a fluid of a green or yellow colour and bitter savour, a jaundice hue about the lips, nose, and on the conjunctiva, yet without fulness or tenderness of the right hypochonder (the absence of which last signs is the proof that the affection has not yet passed into inflammation), we are to conclude that we have to deal with the first degree of hepatic irritation. If it be objected, he further remarks, that hepatic irritation, far from being accompanied with an increased, is often characterized by a suspended secretion of bile, his answer is that the latter is never the case until the irritation has risen into some degree of phlogosis.

Now, with all deference to M. Bonnet, there is not, we apprehend, much novelty in the above statement. We believe we could point in a variety of authors to views essentially the same. We are sure, moreover, that the distinction which M. Bonnet sets forth as novel is, whether or not expressed in so many words, present to the minds of most practitioners, and that it generally regulates practice. We cannot discover wherein "the first degree of hepatic irritation" differs materially from what is ordinarily called functional derangement of the liver, manifesting itself by excessive biliary discharges whether in vomiting or stool. We admit that M. Bonnet, in pointing out that the suspension of the bile in such cases does not take place until some degree of phlegmasia has set in, and is of course symptomatic of the disease having reached that stage, draws a distinction more definite perhaps than is usually done, and one that is useful in diagnosis. Still, we repeat, there is no material novelty in it.

M. Bonnet gives it as his opinion that the majority of practitioners, both in their works and practice, have, till now, supposed themselves to be treating pure parenchymatous hepatitis, while, in fact, their own descriptions show that they have in many cases had a complicated affection to deal with, namely Gastro-Hepato-Peritonitis, as M. Bonnet designates it. He imagines, as we shall see in the sequel, that parenchymatous hepatitis, as a simple and primitive affection, is of rare occurrence; that it seldom exists uncomplicated with gastro-enteritis, peritonitis, or encephalitis. He thinks that a survey of symptoms will, in general, prove the above complicated character of many cases which are set down as those of simple parenchymatous hepatitis; and believes himself to be able accurately to distinguish, amid the *tout ensemble* of symptoms, those referrible to the stomachic and peritoneal affections, from those directly due to the affection of the substance of the liver.

The symptoms, viewed generally, of Gastro-Hepato-Peritonitis are as follows: tension of the right hypochonder; sensibility to pressure; pain sometimes dull, profound, answering ordinarily to the right hypochonder, but having its seat occasionally in the epigastrium or the left hypochonder, and accompanied with a feeling of uneasiness, fulness, suffocation; sometimes acute, pungent, lancinating, analogous to that of an inflamed pleura, and extending in certain cases from the extremities of the right false ribs to the clavicle and arm of the same side; augmentation of the volume of the liver; decubitus difficult, sometimes impossible, now on the one side, now on the other; dyspnœa; respiration increased on the left side, diminished on the right, and null in the abdomen; dry cough, hiccup, nausea, vomiting, intense thirst, bitterness of mouth; tongue red on its edges, and covered in its middle with a yellow or green fur; commonly a yellow tint of the eyes and of the skin; constipation; white stools, or else superabundant secretion of bile, more acrid than in the normal state; urine yellow, scanty, having an oily appearance, depositing a brick-like sediment; skin dry and burning; finally, a pulse frequent and often hard, but in some cases unequal and even intermitting.

Such are the conjoint symptoms of the complicated affection Gastro-Hepato-Peritonitis. Amongst these, those referrible to peritonitis are: tension of the right hypochonder; the sensibility to pressure; the acute, pungent, lancinating pain, extending to the clavicle and arm of the same side; difficult or impossible decubitus on the *right* side; there spiration diminished on the right side and null in the abdomen; the dry cough and the hiccup. Those referrible to the gastro-enteritis are the nausea, vomiting, intense thirst, redness of tongue, dry burning skin, frequent and often hard pulse. Those directly due to phlegmasia of the hepatic parenchyma remain, namely, dull, deep pain, answering generally to the right hypochonder, but having its seat sometimes at the epigastrium or left side, accompanied with a feeling of uneasiness, fulness, suffocation; decubitus difficult or impossible on the *left* side; bitterness of mouth, yellow tongue, jaundice-tint generally of eyes and skin; either white stools, or more or less abundant and acrid bilious ones; urine yellow, scanty, oily in its appearance and depositing the brick-like sediment. M. Bonnet believes justly that parenchymatous hepatitis never advances far without involving the above complications and giving rise, of course, to the above symptoms.

We do not hesitate to admit that it is practically important to ascertain, as far as possible, whether and in what degree the peritoneal covering and the gastro-enteric mucous membrane participate in any primary affection of the substance of the liver, as thereby the prognosis and the treatment must be very considerably modified. We also acknowledge the accuracy with which M. Bonnet classifies the symptoms of the complicated affection, and refers them to the respective lesions which give rise to them. But we are disposed in this, as in the former case, to suspect that M. Bonnet underrates the discernment of his medical brethren, when he supposes that they are in the habit of overlooking the peritoneal and gastro-enteric complications of hepatitis, and of believing they have pure hepatitis to contend with, when the complications referred to are also present. The superficial acute tenderness which exists when the peritoneal coat of the liver is inflamed cannot be misinterpreted by any attentive physician; the gastro-enteric irritation, in all cases of acute hepatitis, is too prominent to escape the remark of even the careless. Not only the possibility but also the high probability of such complications cannot fail to be present to the mind of every practitioner of the slightest experience.

M. Bonnet distinguishes, as has been usually done, two forms of hepatic irritation, the primitive and the consecutive. Of the primitive form, the only causes which he admits are "a blow; a fall on the right hypochonder; a penetrating wound of the abdomen; a violent shock in the vertical line of the body, such as happens from a fall on the feet, hips, or knees." (p. 197.) However, in the succeeding page, he seems to acknowledge, if we understand him aright, other causes of primitive hepatitis.

"It occasionally happens," he says, "that under the influence of an obstacle to the circulation, situated in the chest or in the abdominal cavity, the blood accumulates in the liver and distends it beyond measure. The congestion is here entirely mechanical; but one easily perceives that if such congestion persists or is often repeated, it may become a cause of inflammation to the tissue in which it has its seat. Ought hepatitis of this origin to be regarded as idiopathic? Some will be surprised that I reply in the affirmative; but I wish them to observe that the only intelligible course in etiology is not to regard any morbid affection as consecutive to another, except when the latter has produced it directly. Now, it is not the obstacle to the circulation which, in the case in question, occasions, properly speaking, the hepatitis; but, in fact, the blood, which, accumulated in the liver, becomes by its presence a cause of stimulation to that viscus; the immediate cause of the evil resides in the parenchyma itself. Why, therefore, should not hepatitis, which developes itself in this manner, not be regarded as primitive or idiopathic?" (p. 198.)

Granting it to be so (although the distinction seems more theoretical than of any practical utility), still primitive hepatitis of this origin does not come within range of any of the causes immediately before assigned by M. Bonnet himself. In the above statement, however, of the causes of primitive hepatitis, we do not find much to object to. Let us now see what are the only causes which he recognizes of the consecutive affection. "Consecutive hepatic irritation," he remarks (p. 199), "is always the result of a gastro-enteritis, an inflammation of the peritoneum, or of an encephalitis."

His own illustrations of this theory are not on the whole very clear and satisfactory. We see that he admits only three causes of hepatic irritation. Of these, he considers very justly gastro-enteritis as by far the most frequent. It is through the medium of this last affection, indeed, that he supposes one of the other two, namely, encephalitis, very frequently operates on the liver; and through the same medium, he believes that a multitude of other remote morbid influences or affections act in producing hepatic irritation.

"Dark and fat viands," he remarks, "............ hot seasonings, as pepper ........ generous wines, spirituous liquors, emetics, and purgatives employed continuously; all the agents, in short, known under the name of stimulants, exert their first effect on the stomach and duodenum. *It is never, except when they have produced a gastro-duodenitis*, and that this last has transmitted itself to the liver by the biliary passages or by the veins which originate on the internal surface of the mucous membrane, that the signs of hepatic irritation manifest themselves." (p. 206.)

This view of the matter is certainly just so far; as we believe that a gastro-duodenitis never occurs without inducing irritation greater or less of the liver; but we think M. Bonnet errs in supposing that unduly rich, fat, and stimulant aliment exerts a deleterious influence on the function of the liver in no other way than by the medium of the gastro-duodenal mucous membrane, and the production of a gastro-duodenitis. Aliment of the kind referred to, is generally considered, and we believe justly, as capable of affecting the function in another and indirect manner, namely, by giving such a constitution to the circulating fluid, as, by augmenting the eliminatory duty of the liver, shall derange that organ.

In short, it may here he observed generally, that M. Bonnet makes no reference to the liver as a depuratory organ, nor notices in any part the possibility of some of its derangements being due to lesions of its depuratory function, induced irrespectively of any mediate affection of the stomach and duodenum. We conceive that this omission constitutes a more remarkable and serious "lacune" in his treatise than the one which he refers to at page 222 does in the work of Broussais. The same anxiety to generalize as much as possible the causes of consecutive hepatic irritation, and the same neglect to take into consideration the depuratory function of the liver, and the antagonist or rather supplementary relation which the organ very evidently bears to another important secreting and excreting viscus, namely, the lungs, lead him to represent the biliary affections of tropical climes as also solely owing to a prior affection of the digestive organs.

"Most authors," he observes, "have maintained that residence in a hot climate is one of the conditions which contribute remarkably to the development of diseases of the liver. It is true that such affections are exceedingly common in certain countries, as Egypt and the East Indies; but this is not owing to heat affecting directly the organ which secretes the bile. The most ordinary effect of elevated temperature is to render the digestive passages very excitable, and to dispose peculiarly to gastro-intestinal irritations. These irritations once declared propagate themselves to the neighbouring tissues; hence the frequency of the hepatitis of warm countries." (p. 204.)

Now, the experiments and observations of physiologists of an earlier period, and more lately those of Autenrieth, Tiedemann, and Gmelin, to which M. Bonnet, and we think Dr. Thomson also, makes no reference, tend to show that there is a vicarious action between the liver and the lungs. They even seem to prove that the size of the former organ is, if not always at least generally, in the inverse ratio of that of the latter, making it extremely probable that they are designed and adapted, to some extent at least, mutually to supplement each other. The liver, it is known, eliminates from the blood the excess of certain matters principally composed of carbon and hydrogen, and also fatty matter. The lungs also free the circulation from substances containing a great proportion of carbon. It is true that as regards the liver the carbon is still, when excreted, in the oxidizable state, and when separated by the lungs is united with oxygen; but this seems to prove nothing against the fact of the two organs being vicarious or supplementary in certain cases. Now it has been ascertained that, in states of high atmospheric heat, the lungs excrete less carbon than usual. It seems exceedingly probable that as, in the same circumstances, the action of the liver is augmented, the latter acts vicariously of the lungs; that thus, its function may be increased independently of any affection of the digestive organs; and as, according to M. Bonnet's own showing, undue augmentation of an organ's function cannot take place without irritation, hence one way, not referred to or accounted for by him, in which the liver may be consecutively deranged.

We have stated that the liver also excretes fatty matter. Now we would, in part, account for the frequency of biliary affections in *gouty* subjects, from the constitution of the circulating fluids peculiar to such subjects, and not from a "repercussion" of the gout on the digestive organs and thence on the liver.

"A chill," observes M. Bonnet, "from exposure to a current of air or immersion of part or the whole of the body in cold water, the repercussion of an exantheme, of gout, or of rheumatism, are capable, as we have said above, of acting directly on the liver; but most commonly when inflammation of that organ ensues from sudden suppression of cutaneous perspiration, the disappearance of an exantheme, &c., these causes begin by exciting either a gastro-enteritis or a peritonitis, according as the patient is predisposed to the one or the other of these maladies, by the state of the atmosphere, his regimen, habits, constitution." (p. 207.)

We are of opinion that several of the causes here referred to do not operate on the liver in the manner supposed by Bonnet, but by certain changes produced in the constitution of the blood, by which the excretive function of the organ is affected. In the above quotation, M. Bonnet speaks of the repercussion of an exanthema, of gout, rheumatism, &c. being capable of "acting directly on the liver," and afterwards he makes the following observations:

"Excess of study, violent passions, profound chagrins, accesses of anger, insolation, do not act directly on the liver. All these causes commence by producing an irritation of the brain and its membranes; this, when it is intense or long continued, reacts sometimes *sympathetically on the hepatic parenchyma itself;* but most frequently it transmits itself to the gastro-intestinal mucous membrane, and it is only after this that hepatitis takes place." (p. 213.)

There are two points connected with the above statement which M. Bonnet leaves unexplained, but respecting which we should have wished much to have had some precise enunciation of his views. The first is, in what manner and in what circumstances the repercussion of exanthems, gout, rheumatism, &c. "act directly on the liver;" in short, we should like to know what, according to M. Bonnet's understanding of it, are the meaning and amount of these words. The second point is, how irritation of the brain and its investments acts sympathetically on the liver. It is obvious, from M. Bonnet's words, that he supposes such irritation may and sometimes does so act, and this independently of any mediate affection of the stomach and duodenum. Besides that we are of opinion that M. Bonnet's omission to point out, in a satisfactory manner, the channel and mode of these morbid influences and effects constitutes a radical defect in his theory, we must state it as our impression that he has himself supplied the refutation of his own hypothesis, by admitting that there are several ways in which hepatic irritation may be originated other than the three which, at the outset, he stated as the sole ones: to wit, a gastro-enteritis, a peritonitis, or an encephalitis.

The view that many derangements of the liver are owing to previous error of the gastro-duodenal mucous membrane is not a new one. Broussais, we know, had, long before Bonnet, announced and insisted on it. Bonnet himself admits this:

"Such are my ideas of the mode in which hepatic irritation is produced. They approach nearly, I own, to those of Broussais on the same point; but they are not identical, as he pretends in his Annals. .......... Independently, in fact, that this physician has not recognized any degree of irritation of the liver, but that which constitutes the hepatitis of authors, he says nowhere in his writings that this last may be caused by peritonitis." (p. 22.)

Now we have two remarks to make here. In the first place we doubt that a genuine hepatitis, involving the substance of the liver, is ever due to a peritoneal affection, except in cases in which the liver has been previously the seat of chronic disease, and even of some degree of structural change; and in which also, the person in whom it occurred was of a peculiarly cachectic diathesis. We have never ourselves seen any case of peritonitis, however grave and even though terminating fatally, in which it could be affirmed that the hepatitis complicated with it was purely consecutive of the peritoneal affection. No doubt, when the peritoneal investment of the liver itself is the seat of inflammation, a superficial affection of the parenchyma usually follows; but not a general and profound hepatitis. Secondly, we do not see that M. Bonnet has any ground for representing as novel the fact that peritonitis may, in the degree and in the circumstances above indicated, induce hepatitis; nor can we admit that Broussais was not cognizant of such a contingency; for although that eminent man may not have stated the possibility of such a complication in so many words, it is very clear, from various parts of his writings, that in respect to him, it could not be called new or unknown.

In the second part of his treatise, M. Bonnet treats of what he calls "maladies of the liver, other than hepatic irritation,"—of which we shall presently give a brief sketch or rather enumeration. Meanwhile we shall follow the author's plan, and advert to the treatment of hepatic irritation.

Whatever opinions we may entertain of some of the theoretical views of M. Bonnet, we must acknowledge that we regard his treatment as simple, sound, and efficacious. We cannot do better than give it in his own words. It will be seen that it is neither, on the one hand, so tame as the practice of his countrymen sometimes is, nor, on the other, so rash and bloody as are the measures of some medical men on this side of the channel. After observing that in cases in which inflammation of the liver depends on a lesion of the digestive passages the best way is to attack the two affections simultaneously, he continues:

"In all cases in which acute hepatitis depends on a gastro-enteritis, and the subject is plethoric, we should commence the treatment by bloodletting from the arm. If, in spite of this depletion, the pulse remains large, hard, and full, we must repeat it, and then have recourse to local emissions. These last suffice almost always with persons who are neither plethoric nor strong, or whose phlegmasia is not intense; but it is necessary, even in such cases, that the emissions be abundant and often repeated. We are in general too reserved in our employment of them. . . . . We must enforce the most severe abstinence, and prescribe cold acidulated drinks, as lemonade, &c." (p. 229.)

He recommends the simultaneous employment of clysters to which nitrate of potass has been added, then baths, and gentle purgatives.

We should in this country reckon the above treatment too mild perhaps for many cases of hepatitis. It is evident that Bonnet is swayed by the same groundless apprehensions which so fettered Broussais in regard to the supposed hazard of exhibiting purgatives on an excited mucous membrane. Yet apparently he is aware of the good effects of purgatives, for in reference to their employment in chronic hepatitis, he remarks:

"The English, who make very little enquiry as to the condition of the digestive passages, and who give to every patient, without exception, calomel, alone or combined with aloes, gamboge, &c. effect, if we are to believe them, many cures. There is a slight exaggeration in what our neighbours on the other side of the channel tell us of their practice in this respect; but it is certain that they often cure. Now although, among their successful cases, there may be some which ought to be referred to the efforts of nature or the absence of gastric irritation, there are others which are really due to the action of the medicine on the inflamed stomach; and one cannot but admit that stimulants which act by astringing (resserrant) the tissues, or which provoke an abundant secretion of mucosities from the surface of the gastro-intestinal mucous membrane, sometimes dissipate both the irritation of that membrane, and the hepatitis itself. There can be no doubt in my opinion as to this." (p. 259.)

Yet immediately after thus expressing himself, he says of mineral waters : " taken internally, their only operation is to stimulate the gastro-intestinal mucous membrane. . . . . It is scarcely possible, theoretically speaking, to depend on their efficacy." We differ very decidedly from M. Bonnet in this view, and we find that, by his own admission, Bordeu, Portal, Bouvard, and many other distinguished physicians had prescribed these waters with great benefit.

We would call attention to Bonnet's mention of baths in the treatment of hepatitis. Portal seems to have employed them with advantage; and there is no doubt that in hyperemic states of the liver and of the abdominal organs in general, baths are of the greatest efficacy as auxiliaries to other means,—as, by encouraging superficial circulation, they relieve central congestion. It is, however, a singular proof of M. Bonnet's ignorance or disregard of the modes of treatment followed by practitioners of other countries, that he makes no mention, so far as we can discover, of the nitro-muriatic acid bath; of the remarkable good effects of which in certain cases of chronic hepatitis and functional derangements of the liver, there can be no doubt; notwithstanding the doubts which Dr. Thomson (p. 290 of his Practical Treatise) is pleased to throw out on the subject.

M. Bonnet thinks leeching at the anus has no special efficacy whatever in relieving the hepatic venous system, and thinks it indicated only in cases in which hepatitis has supervened on the non-appearance or suppression of a periodical hemorrhoidal discharge, or of menstruation. On this subject many eminent practitioners differ from him. Dr. Conwell remarks that " local depletion is most efficaciously performed by the application of leeches round the anus, over the abdominal parietes and the perineum, as the blood drawn from those parts reduces the quantity about to be poured directly into these sources of the portal system, whether it be derived from arterial or venous capillaries." (Treatise on the Liver, sect. 185.) We have ourselves seen great obvious benefit in many cases from the application of leeches about the anus; but, in order to this effect, the number of leeches applied must be considerably greater than it frequently is.

Bonnet is also opposed to the employment of cupping-glasses and blisters, which, he remarks (p. 232,) " if not always hurtful, are at least perfectly useless in the treatment of acute hepatitis." We know Mr. Annesley thinks that cupping-glasses, by the tightening of the integuments and the pain, which in this way they cause, are apt to augment the inflammatory action, and that various eminent practitioners are opposed to the use of epispastics, especially of cantharides, in acute hepatitis. But we must confess that we have not by any means so unfavorable views of these remedies as M. Bonnet seems to entertain. We have seen the greatest benefit, even in acute hepatitis, from the application of cupping-glasses. As to blisters, we should certainly not have recourse to them in this form of hepatitis, both because their use in it is questionable, and their presence might prevent the application of the more direct and less equivocal means, leeching and cupping. But in some forms of chronic hepatitis their good effects are unquestionable.

The remainder of M. Bonnet's work is occupied with the rarer affections of the liver; and we do not find that, in treating these, he has advanced anything remarkably new or important; although his observations in general are interesting and intelligent. We shall enumerate a few of the "diseases other than hepatic irritation." These are, hepatic asthenia, atrophy of the liver, passive sanguineous congestions of the organ, hepatic hemorrhages, erectile tumours, hepatic phthisis, biliary calculi, hydatids, hepatic tumours, jaundice, diseases of the excretory function of the liver.

Hepatic asthenia consists "in a debility greater or less of the biliary apparatus," (p. 273,) in which, "the quantity of bile secreted is too little to stimulate efficiently the intestinal mucous membrane." M. Bonnet seems to have found pills of aloes, calomel, and scammony useful in this affection.

The "hepatic phthisis," remarks M. Bonnet, (p. 307,) "of our predecessors is nothing else than chronic hepatitis, which has lasted long, and produced wasting, consumption, and death. It is, therefore, perfectly useless to constitute it a particular morbid state, or to enter into details respecting it."

In respect to jaundice, M. Bonnet is disposed to regard all the modern theories as either false or at least not proven, and as furnishing uncertain grounds of practice; such as that this malady is due to a dissociation of the elements of the blood, or to a morbid constitution of that fluid, or to the liver not duly separating from the circulation the biliary principles. He thinks the view which the ancient physicians took of jaundice, as the result of some positive obstruction to the excretion of the bile, and the consequent absorption of that liquid into the sanguineous circulation, the only or, at least, the most sure and the most trustworthy, as a ground of treatment. And undoubtedly M. Bonnet has reason in thinking that, in almost every case of jaundice, it is safest for us to *assume* that the excretion of the bile is obstructed by some mechanical cause, and to direct our practice accordingly. At the same time, while we admit that our knowledge of the causes of jaundice, other than gallstones or some mechanical or structural impediment in the biliary passages, is still imperfect, we think there are sufficient reasons for believing that the disease is occasionally owing to some morbid change in the constitution of the blood, or abnormal action in the secretory function of the liver, in consequence of which, matters which should have been eliminated from the circulation are retained in it.

In M. Bonnet's concluding section, "alterations of the bile," there is nothing worthy of remark.

We pass now to the Treatise of Dr. Thomson. Much less original in its character than that of Bonnet, it contains much more information. It is, in truth, a most laborious and complete compilation, but nothing more; everything valuable and important comprised in it existing already in print. We do not recollect a single observation, doctrinal or practical, of the slightest moment, which Dr. Thomson is not careful to corroborate by some quotation from a previous writer; thus himself showing that the sole merit of his work lies in being a collation and digest of the opinions and practice of various writers on liver disease.

As Dr. Thomson, though following a different method, goes over nearly the same ground as Bonnet, as he seems to draw no small portion of his materials from this writer, and as, agreeably to the remark just made, the greater part of his book is but a rehearsal of opinions and practice already before the public, we do not deem it requisite to enter on a minute examination of his volume, but shall simply advert to one or two points which seem to require special notice.

In the preface Dr. Thomson remarks, that "he thinks it right to mention that the opinions relative to the employment of mercury in affections of the biliary organs, which he has endeavoured to illustrate and support, have long been entertained by his father, and guided his practice in this class of affections: and that they were inculcated by him in the course of lectures on the practice of physic, which he delivered for several sessions in the Edinburgh School of Medicine." We do not know whether Dr. Thomson means to claim for his father the having propounded some new views as to the employment of mercury in liver complaints, or simply to adduce his father's eminent authority in favour of certain views which had been held by others before him. We must infer that the latter is the meaning of Dr. Thomson, since he himself remarks, page 253 of his Treatise: "It is but justice to the late Dr. Trotter to observe that, in his view of the nervous temperament, he was one of the first physicians in this country who opposed the fashionable practice of administering mercurial medicines in the treatment of the so-called bilious complaints. In that opposition he was ably seconded by Dr. Saunders." Now the third edition of the latter gentleman's work on the Liver was published in 1803, and his Observations on the Hepatitis of India, which contain the cautions referred to in respect to the use of mercury, were published in 1809. But besides this, we confess ourselves at a loss to discover what are the peculiar opinions to which Dr. Thomson inclines with regard to the use of mercury, for he summons almost equally respectable authorities on either side of the question; and is careful (a peculiarity which pervades his book) to give nothing but a most negative and indefinite opinion of his own. In short, the whole of his section on mercury consists in quotations, alternately of writers who are of opinion that mercury is principally to be relied on in the treatment of hepatitis, and of others who take a more qualified view of its use and expediency; and although we may gather, though not without difficulty, from the general strain of the section, that Dr. Thomson is disposed to side with the latter authorities, yet nowhere do we find him giving that clear expression of his views and practice which the formal announcement in his preface had led us to expect. At page 294, Dr. Thomson declares "we shall not take upon ourselves to affirm in what cases of diseases of the biliary organs, mercury ought or ought not to be had recourse to," &c. If he is not prepared to do so, and if his views are so undetermined as to the powers of mercury and the morbid exigencies which indicate it, we should think it was hardly necessary for him to have asked particular attention to his opinions respecting these points.

It would be out of place to enter here on a minute description of the merits of mercury in hepatic derangements. We freely admit that it is an agent which has been much abused: but while we concede thus much, we must express our conviction that as a cholagogue drug—as a means of overcoming that excretory torpor of the liver in which the bile is duly secreted, but not emulged, in which the biliary tubes and receptacles become distended, (a condition which if allowed to continue may give rise to hyperæmia and congestion, and ultimately inflammation,)—there is nothing so valuable as mercury. When there is reason to suppose that pale-coloured stools are owing to a mechanical cause, as a gallstone or some structural change in the ducts, mercury, as a *cholagogue*, is of course out of the question; and in acute hepatitis,—although as a purgative it is not contra-indicated, but is, on the contrary, combined or followed by saline draughts, &c. highly useful,—no certain advantage is to be expected from it as a constitutional means. In this case, the ordinary antiphlogistic treatment, by bloodletting, local and general, by saline purgatives, acidulous drinks, and abstinence, is alone or chiefly to be trusted to; and mercury, administered so as to affect the mouth, far from benefiting, appears sometimes positively to exasperate the inflammatory action. This seems to be the opinion of most practitioners, and of those in particular whose experience of hepatic cases has been the most ample. After suitable depletion by the lancet, mercury is useful in restoring suspended secretion, whether of the liver or of the intestinal canal, and in thereby still further subduing inflammatory action, or in preventing a reaccession of it.

It appears to us that there can be few members of the profession who practise it with more desponding feelings and anticipations than Dr. Thomson. The cases are rare indeed in which he does not emit sceptical opinions as to the efficiency of the modes of treatment to which he adverts. And thus, in regard to mercury, he remarks, page 279, in the course of some observations as to the power which this medicine possesses over the structural alterations of the liver, "With regard to mercury, as to other resolvents, and perhaps all other medicines, it may fairly be questioned whether the practitioner ever effects by its means what nature never succeeds in accomplishing for herself—whether any of the structural alterations of the liver, not of an inflammatory character, ever disappears under, or in consequence of, its administration or use." We are of opinion that there is no reason to doubt that, whether by exerting some power on the absorbent function, or by removing capillary congestion, or by modifying in some way the action of the secreting vessels, mercury does possess an unequivocal influence over some sorts of morbid formations in various textures and parts more accessible to our observation than the liver; and therefore that this fact, along with other strong presumptive proofs of its being occasionally efficient in reducing enlargement along with sensible induration of the organ in question, makes it extremely probable at least, that it is sometimes successful in removing structural changes of the liver. We are quite willing to admit that mercury is, in all such cases, only an auxiliary to a greater power; or, as Dr. Thomson expresses it, that "there are salutary changes which nature occasionally accomplishes for herself, or with such aid only as is required to give fair play to her own efforts;" but the same may be said of every medicine and of every case in which medicine is had recourse to; and it is moreover to be observed that if, without the aid referred to, nature would have been inadequate to bring about those salutary changes, the agent which enables her to do so, or which removes obstacles thereto, must be regarded as playing an essential part in the fortunate process. We do not suppose that any intelligent man ever attributed more to medicinal agency than what we have stated; but surely it is going beyond the limits of philosophical doubt to deny thus much to it: "By alteratives," says Müller, "an organ morbidly changed in composition cannot be rendered sound by, as it were, a chemical process; but such a slight chemical change can be produced as shall render it possible for nature to restore the healthy constitution of the part by the process of nutrition." It is not to be denied that occasionally mercury seems to accelerate the progress of morbid formation, and to give it a more malignant character. This happens in cases in which the disease is originally of an intractable nature, or in which the constitution or diathesis of the patient is cachectic; and mercury in such cases, no doubt, seems but to facilitate the deposition of the morbid growth.

We shall only remark, in conclusion, that our French neighbours are not now so scrupulous as to the use of mercury in the treatment of hepatic disease as Dr. Thomson appears to suppose. Bonnet seems frequently to employ it, as Portal had done before him, and as Andral now does. Its use is, moreover, becoming every day more common in France. We would also here remark that, as to those cases referred to by Dr. Thomson, in which iodine, nitric acid, &c. were supposed, even by Twining, to augment the hepatic derangements which they were meant to cure, we have ourselves observed that it was only when imprudently prescribed that these substances irritated the gastro-duodenal mucous membrane, and that the hepatic derangement followed; but in no case, in which the former mishap did not take place, did the latter manifest itself: we consequently infer that we may persist in the use of iodine and nitric acid, without fear of injuring the liver, so long as the stomach and duodenum betray no signs of suffering.

Dr. Thomson's work, although well written, reads rather tamely, from the absence of original observation and vigorous opinion. We think that an author, even when he professes merely to compile, ought to digest, sift, and decide among the conflicting statements which he quotes, and not to leave this task to the reader. The principal merit of Dr. Thomson's volume is, as we have already stated, that it gives an accurate *coup d'œil* of facts and doctrines already in print, but scattered through a variety of volumes.

We purposely omitted to notice in their place M. Bonnet's remarks on hepatic abscess and its treatment, (p. 182,) in order that we might refer to them in connexion with some very interesting cases of exploratory puncture, detailed in the second volume of the Madras Medical Journal: a work which exhibits in a most favorable light, the scientific knowledge and professional zeal of our brethren in India, and does great credit to all concerned in its establishment and maintenance. Bonnet gives several successful cases of the evacuation, by operation, of hepatic abscesses: the case mentioned at page 237, in which an abscess of the liver first burst into the lungs, and again forming, was opened from the abdomen, and in which the patient recovered, is an exceedingly rare and remarkable one. As to the propriety of puncturing an abscess of this organ, of the existence of which there is no doubt, and which points in some accessible part, there cannot be the slightest hesitation. But as exploratory puncture supposes that when it is performed, there is some degree of doubt as to the existence and the site of the abscess, and as consequently some practitioners may have scruples, whether in such circumstances, the operation is warrantable and safe, we shall endeavour to estimate what points the cases referred to appear to establish.

In the first place, the operation is in some cases not only safe but successful, and obviously the means of saving the life of the patient. It was so in the case detailed by Dr. Everard. (Journal, p. 229.) Although "there were evidently no adhesions between the liver and the abdominal parietes ;. . . the deputy-inspector . . . thrust a trocar into the liver through the epigastrium, without waiting to make any preparatory operation to induce adhesions between the parts." In another case of a private soldier, detailed by Dr. Mouat, (Journal, p. 226,) the patient "was twice punctured. The first operation failed to reach the cyst, but the second puncture did; when a trocar was introduced and a large abscess evacuated." This man and another on whom the operation was performed, died ; but in both cases, no bad consequences ensued from the use of the trocar ; no effusion either of blood or matter had taken place into the abdomen ; and death was owing to the presence of several other abscesses. There is every reason to presume that in the former of these cases life would have been preserved had the abscess which was punctured been the only one. Regarding the general merits of the operation, Dr. Murray (p. 238) remarks :

"I consider that with a good anatomical, pathological knowledge of the region (i. e. of the liver) in our mind's eye, to enable us to avoid the large hepatic vessels, the gall-bladder, the colon, and the stomach, there is abundance of evidence to authorize us to, nay, it is our bounden duty to explore the liver, without hesitation or delay, in most cases where pathognomonic symptoms of abscess in it exist. . . . . By early accurate diagnosis and active constitutional treatment, a favorable termination may very often be happily brought about in hepatic diseases, without the necessity of operative procedure; but when abscess has once formed, we know how little advantage is to be expected from persisting in the use of mercury or any other medicine. Therefore let the question be fairly put, does not the trocar with a regulated diet hold out a better prospect of success ?" (Journal, p. 472.)

We, however, think M. Recamier's mode of exploration much preferable to the one adopted in the above cases. This practitioner punctures, in the first instance, with a tube of a capillary fineness, over which he places a cupping-glass, by means of which he draws forth some of the contents of the tumour, and ascertains of what nature these are. We are not aware that Recamier is accustomed to adopt even this cautious mode of procedure, except in cases where there is an obvious and defined tumour ; nor do we know that he employs it in the purely exploratory manner of some practitioners in this country, and exemplified in the cases detailed above. But it is obvious that his method is perfectly applicable to all such cases, and would certainly tend to render the operation more safe and sure.

"All our punctures," says Dr. Murray, "should be made from the abdominal cavity,—entering the trocar or explorer under the edge of the cartilages of the seventh, eighth, or ninth ribs, as circumstances may indicate. We may often indeed get nearer to the abscess through one of the intercostal spaces : *and I think primary exploration may sometimes be advantageously made in this situation by a very minute flat canular instrument ;* but from not having seen any patient recover where the matter was evacuated in this direction (through the diaphragm); from finding that the action of the fibres of the diaphragm impedes the free discharge of the matter, something like a valve; from observing that air sometimes enters the wound when made there; and from considering that the opening is not so dependent through the walls of the thorax, as when made through the abdominal parietes,—I beg to recommend the latter mode in all cases." (Journal, p. 239.)

At page 22 et seq. of the Journal from which we have been quoting, Dr. Mouat discusses, at some length and in relation to hepatic abscess, the alleged occurrence of purulent metastasis, and of discharges of pus by stool, by the kidneys, and by the lungs. He gives a table of 112 cases, of which it is asserted that "109 passed purulent matter by urine; 33 of the number also by stool; and 8 of them pus by expectoration." Dr. Mouat, however, immediately after wisely puts the question: "Were these discharges really pus? And, if purulent matter, how can we account for its appearance in the excretions? For the former we have no unerring or certain test." Subsequently, Dr. Mouat states it as his opinion that, as far as his observations extend, "the appearance of the purulent matter seen in the fæcal and urinary discharges had passed through the circulatory mass." The possibility of the removal in this way of the pus of abscesses was and is held by Morgagni, Petit, Legallois, Larrey, Velpeau, Hennen, Copland; and has been variously explained and accounted for. Hunter denied it: and later researches and more accurate anatomical observation seem to prove that, unless where there is a direct communication between the abscess and the mucous surface, whether intestinal, pulmonary, or vesical, from which the purulent discharge takes place, no such discharge can occur; that it is impossible that the pus of an abscess can pass through the capillary circulation and then be excreted from a mucous surface: and that the alleged purulent discharges of this kind were either such only in appearance, or, if truly purulent, directly proceded from the organ or surface itself on which they showed themselves. This view is founded on very strong and as it seems to us incontrovertible evidence. Not to refer to the negative fact that Andral, a careful observer, has never, notwithstanding repeated and anxious examination, detected pus in the lymphatics in the neighbourhood of abscesses, but what was the product of inflammation in these lymphatics themselves, it has been ascertained by Weber that the globules of pus are always large, and in general are twice as large as the red particles of the blood, and therefore the former cannot possibly pass by the same routes as the latter. Moreover there are no apertures in the walls either of the lymphatics or of any of the secreting mucous surfaces, so large as to admit of the globules of pus, either by entering or escaping thereby; so that it is in the first place impossible, except mechanically, namely, by some rupture or ulceration in the walls of the lymphatics, that pus can find access into these vessels; and, in the second place, it is equally impossible, even after they have thus got into the circulation, that they can pass the capillaries, or, even supposing this obstacle overcome, that they find exit from any secreting or excreting mucous surface, except by a lesion of that surface of the most serious kind. We have only to add that the official reports, as given in this Journal, of the present mode of treating hepatic affections in India do not countenance the opinion, which Dr. Thomson's statements seem to imply, that mercury is less confided in than formerly. We do now not enquire whether the employment of that agent, to the extent in which it is there used, is proper and defensible or the reverse: but that it is still principally trusted to may be learned from such statements as the following:

"If the original inflammation of the liver be slight, then moderate depletion and mercurialization will effect the solution of the circulating clot, and also the effused clot in the inflamed organ, and consequently remove the obstruction to the inflammation in the inflamed organ. Again, in very acute inflammations, the tendency of (to ?) adhesion in the circulating blood, and the effused clot is so great, that copious depletion and mercurial saturation are required to overcome it. . . . In slight attacks, mercury has speedy success, and this is accompanied by glandular salivation. . . . . After admission, when it was detected that the liver was affected, bleeding and leeching were had recourse to, with large doses of calomel, sometimes amounting to a scruple, sometimes to ten grains. . . . . The quantity of blue pill and calomel, when calculated, was often large altogether," &c. (Madras Medical Journal: July, 1840. Art. I. Medical History of H. M. 39th Regiment, &c.)

---

